# Research progress on the molecular mechanisms of chlorogenic acid’s pharmacological effects and its advanced drug delivery systems

**DOI:** 10.3389/fphar.2026.1746172

**Published:** 2026-03-18

**Authors:** Linran Gao, Tianci Zhang, Xianyu Zhang, Wenbo Ding, Jianyu Yang, Yu Luo, Jingjing Wang, Xin Yuan, Yundong Zhao

**Affiliations:** 1 School of Medical Technical, Beihua University, Jilin, China; 2 School of Laboratory Medicine, Beihua University, Jilin, China

**Keywords:** anti-inflammatory, drug delivery, epigenetic, molecular mechanism, pharmacological

## Abstract

Chlorogic acid (CGA), a prevalent polyphenol formed in plants, is recognized for its broad spectrum of biological activities, including potent antioxidant, anti-inflammatory, antimicrobial, andmetabolic regulatory effects. This review synthesizes evidence demonstrating CGA’s therapeutic potential across various conditions, such as hypertension, gout, atherosclerosis, and metabolic disorders. Key mechanisms involve neutralizing reactive oxygen species, modulating critical signaling pathways like TLR2/NF-κB and MAPK, enhancing insulin sensitivity, and influencing endothelial function by boosting NO production via eNOS phosphorylation. Despite these promising attributes, CGA’s clinical translation is hindered by significant bioavailability challenges stemming from poor solubility, rapid metabolism, and instability in physiological environments. To address these limitations, innovative drug delivery systems are being investigated. These systems, including polymeric nanoparticles, liposomes, micelles, and hydrogels, offer strategies to encapsulate CGA, thereby protecting it from degradation, improving its dissolution properties, and enabling controlled or targeted release at specific sites of action. Such advancements in delivery technology are crucial for enhancing CGA’s stability and bioavailability, ultimately bridging the gap between its demonstrated *in vitro* and preclinical efficacy and its realization as an effective therapeutic agent in human medicine.

## 1 Introduction

**FIGURE 1:**
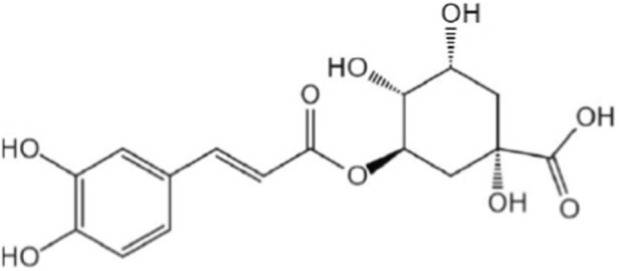
Chemical structure of chlorogenic acid.

Chlorogenic acid, as a core esterified polyphenol formed from the condensation of caffeic acid and quinic acid via an ester bond, possesses a unique molecular structure—comprising a hydroxyl-rich polycarboxylic acid ring (quinic acid) and a phenolic acid side chain (caffeoyl) featuring an α,β-unsaturated system. This structural motif renders CGA a highly valuable and versatile biosynthetic building block (Zheng et al., 2023; Jiao et al., 2024), with the chemical formula C16H18O9 (Figure 1). CGA is widely distributed in various plants, occurring in the stems and leaves of traditional Chinese medicinal herbs such as Eucommia ulmoides and *Lonicera japonica*, as well as in dietary sources like tea, roasted green beans, and coffee (Xiao et al., 2023). Notably, CGA exhibits favorable water solubility; for instance, brewing 4.5 g of chamomile tea yields approximately 72 mg of CGAcan be obtained (Hayakawa et al., 2020). Accumulating evidence indicates that CGA plays a pivotal therapeutic role in the management of various pathologies, including hypertension (Zhu et al., 2022; Agunloye et al., 2019), gout (Chen et al., 2011; Meng et al., 2014), atherosclerosis (Meng et al., 2024), and Parkinson’s disease.

The chemical properties of CGA are relatively unstable, although it is readily soluble in polar solvents such as water, methanol, and ethanol (Jeon et al., 2025). The solubility of CGA is significantly influenced by pH; specifically, under alkaline conditions, CGA undergoes dissociation, resulting in decreased solubility (Drucker et al., 2023). Due to this chemical instability, the application of CGA is somewhat restricted, necessitating conjugation or specific delivery systems for activation in certain contexts. Studies have demonstrated that attaching CGA to soluble oat β-glucan can enhance its stability and potentially augment its bioactivity, thereby facilitating its application in the food industry (Luo et al., 2023). Recent in-depth investigations into CGA have revealed diverse biological activities, including antioxidant (La Rosa et al., 2023), anti-inflammatory (Li et al., 2024), antimicrobial (Le et al., 2022), and antitumor (Wu et al., 2025) effects. Research indicates that CGA exerts significant anti-inflammatory and antioxidant effects by modulating signaling pathways such as MAPK and NF-κB, and regulates blood glucose by ameliorating insulin resistance (Yan et al., 2022). In terms of antimicrobial activity, CGA acts by disrupting cell membranes. Currently, omics technologies are being employed to explore molecular targets, with the aim of confirming the material basis for its multi-faceted therapeutic effects against metabolic syndrome and neurodegenerative diseases (Ma et al., 2014; Wuttimongkolchai et al., 2022). And mitochondrial-dependent apoptotic pathways well-established—the clinical evidence in these fields remains preliminary. Most human studies regarding neuroprotection are currently limited to observational epidemiological studies linking high CGA intake. Subsequent evaluations of its therapeutic potential under relevant pathological conditions have demonstrated promising efficacy. However, there remains an urgent need for further research to fully elucidate the underlying molecular mechanisms involved in these therapeutic effects and their clinical translation.

## 2 Antioxidant effects and mechanisms

Homeostasis in living organisms relies on the balance between oxidation and antioxidant systems (Jones, 2006). When this balance is disrupted in favor of an oxidative state, the excessive production of reactive oxygen species (ROS) can damage biological macromolecules and subsequently induce various diseases (Jones and Sies, 2025). CGA exhibits significant antioxidant properties and can effectively prevent and treat various diseases by modulating the levels of oxidative stress-related enzymes, such as reactive oxygen species (ROS), superoxide dismutase (SOD), and cyclooxygenase (COX) (Remigante and Morabito, 2022). Its mechanism involves multi-level regulation, including the modulation of the endogenous antioxidant enzyme system, direct scavenging of free radicals, and metal ion chelation (Kono et al., 1998).

### 2.1 Modulation of redox-sensitive enzymes and signaling pathways

CGA exerts cytoprotective effects by regulating specific redox-sensitive signaling pathways. In the context of oxidative stress, CGA can activate the Nrf2 pathway, significantly upregulating the expression and activity of downstream target genes (such as GLRX) and key endogenous antioxidant enzymes, including superoxide dismutase (SOD) and glutathione peroxidase (GSH-Px) (Li X. et al., 2022). This enzymatic effect mitigates oxidative damage through two primary pathways: first, SOD catalyzes the disproportionation of superoxide anion (O2•-) into hydrogen peroxide; second, GSH-Px catalyzes the reduction of hydrogen peroxide or lipid peroxides (utilizing reduced glutathione, GSH), thereby significantly reducing oxidative DNA damage and apoptosis (Chen et al., 2022). This mechanism has been further confirmed in vivo studies on the intestinal tissues of piglets, where CGA upregulated the expression of antioxidant genes such as Nrf2 and HO-1, thereby enhancing the body’s antioxidant capacity. Furthermore, CGA demonstrates specific inhibitory activity against pro-inflammatory enzymes, including cyclooxygenase-1 (COX-1) and cyclooxygenase-2 (COX-2), thereby reducing the production of oxidative and inflammatory mediators such as prostaglandins (Hudson et al., 2022; Palócz et al., 2016).

### 2.2 Direct scavenging of free radicals

Free radicals play a crucial role in maintaining homeostasis by participating in immune responses and intracellular communication (Izu et al., 2024); however, their excessive accumulation leads to pathological damage (Ayala et al., 2014). Exposure to outdoor ultraviolet B (UVB) radiation during outdoor activities can induce oxidative damage in HaCaT keratinocytes (Wiswedel et al., 2007). *In vitro* experiments have demonstrated that CGA effectively scavenges DPPH radicals, superoxide anions, hydroxyl radicals, and intracellular ROS. Mechanistic studies reveal that CGA inhibits UVB-induced apoptosis, modulates the expression levels of apoptosis-related proteins (Bcl-2, Bax, and caspase-3), further reduces UVB-induced DNA damage, and enhances cell viability (Bogavac-Stanojevic et al., 2018). Additionally, the accumulation of oxidized low-density lipoprotein (ox-LDL), formed when increased blood low-density lipoprotein (LDL) binds with oxidative free radicals (Wu et al., 2014), leads to the development of atherosclerosis (AS). *In vitro* studies on diabetes-associated atherosclerosis indicate that CGA significantly reduces ROS production and increases the expression of antioxidant enzymes (Nrf2 and catalase) (Yang TY. et al., 2022) by inhibiting the expression and phosphorylation of pathways such as FAK, PI3K/Akt, and NF-κB, and downregulating the expression of Rac1, RhoA, and Cdc42. Moreover, CGA exerts anti-atherosclerotic effects by reducing the migration and proliferation of vascular smooth muscle cells (VSMCs) (Yang TY. et al., 2022).

### 2.3 Chelation of metal ions

The molecular structure of CGA contains multiple atoms capable of providing lone pairs of electrons, primarily oxygen atoms on phenolic hydroxyl (-OH) and carboxyl (-COOH) groups. These oxygen atoms act as ligands, forming coordinate bonds with the empty orbitals of metal ions to generate stable cyclic chelates (Nowak et al., 2022). To deeply investigate the antioxidant capacity of CGA, studies have combined *in vitro* chemical analysis with Density Functional Theory (DFT) calculations. DFT calculations indicate that Zn (II)-CGA complexes possess a higher Highest Occupied Molecular Orbital (HOMO) energy level and lower ionization potential, which enhances their electron-donating capability and antioxidant activity, thereby increasing FRAP and CUPRAC activities (Kalinowska et al., 2020; Kalinowska et al., 2022). Furthermore, comparative studies have found that the CGA-Cu^2+^ complex exhibits higher pro-oxidant activity in the Trolox oxidation assay, and the lipophilicity of the complex is lower than that of CGA alone, which may constitute the structural basis for the difference in their cytotoxicity (Tošović and Marković, 2019).

### 2.4 Clinical application of the antioxidant effects of chlorogenic acid

**FIGURE 2:**
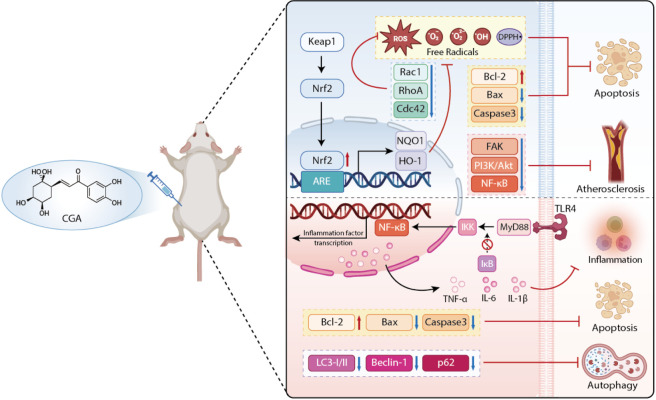
Schematic diagram of the mechanism by which CGA regulates atherosclerosis-related pathological processes through the Nrf2/Keap1 signaling pathway.

The pathological core of atherosclerosis primarily lies in the formation of oxidized low-density lipoprotein (ox-LDL), involving mechanisms such as dyslipidemia, oxidative stress, and immune cell activation, which ultimately lead to vascular wall thickening, decreased elasticity, and blood flow obstruction (Guo et al., 2023). An *in vivo* study utilized ApoE (−/−) mice to establish a high-fat diet model, administering a 12-week intragastric intervention with CGA at doses of 2, 20, and 400 mg/kg, using atorvastatin as a positive control. Plasma biochemical analysis revealed that the high-dose CGA group significantly reduced total cholesterol, triglycerides, and LDL-C levels. Furthermore, pathological examination of the aortic root confirmed that CGA effectively reduced lesion area and improved vasodilation (Wu et al., 2014). Compared to its high efficiency in directly scavenging free radicals and inhibiting LDL oxidation *in vitro*, the bioavailability of CGA in the complex *in vivo* environment is relatively low; however, significant biological effects have still been confirmed in clinical studies (Hada et al., 2020). Clinical research indicates that consuming coffee or extracts containing CGA helps improve vascular endothelial function and lower blood lipids. Additionally, cohort studies suggest that higher CGA intake may be associated with a reduced risk of stroke and all-cause mortality (Ding et al., 2014). Below is a schematic diagram of the mechanism by which CGA regulates atherosclerosis-related pathological processes through the Nrf2/Keap1 signaling pathway (Figure 2).

## 3 Anti-inflammatory effects and mechanisms

The inflammatory response is a defensive physiological reaction against harmful stimuli such as pathogen infections and tissue damage. It functions to eliminate pathogens and repair damaged tissues. However, when the inflammatory response becomes uncontrolled, over-activated, or chronic, it disrupts cellular homeostasis, induces oxidative stress, and leads to pathological tissue remodeling, thereby driving the onset and progression of various chronic diseases, including neurodegenerative diseases, diabetes, autoimmune diseases, and cancer (Jerigova et al., 2023; Xu et al., 2020). Therefore, pharmacological intervention to suppress excessive inflammation is of significant clinical importance. As a natural anti-inflammatory agent, the mechanism of CGA involves multi-dimensional signaling pathway blockade, immune cell function regulation, and modulation of cellular autophagy.

### 3.1 Inhibition of inflammatory signaling pathways

CGA exerts its anti-inflammatory effects primarily by blocking the two classic inflammatory signaling cascades: NF-κB and MAPK.

#### 3.1.1 Blocking the TLR/NF-κB signaling axis

The Toll-like receptor (TLR) family serves as critical sensors for innate immunity (O’neill et al., 2013). Research by Xu et al. demonstrated that in a model of bovine mammary epithelial cells (BMECs) stimulated with *Staphylococcus aureus* lipoteichoic acid (LTA), CGA significantly downregulated TLR2 expression and inhibited the gene and protein expression levels of downstream mediators, including TNF-α, IL-6, and IL-1β, as well as NO production. Furthermore, it inhibited the phosphorylation of NF-κB signaling pathway-related proteins, I-κBα and p65 (Xu et al., 2023). Additionally, in a rat brain injury model, CGA was confirmed to exert neuroprotective effects by inhibiting the TLR4/NF-κB signaling pathway and downregulating the levels of tumor necrosis factor-α (TNF-α), interleukin-1β (IL-1β), and interleukin-6 (IL-6) (Zheng et al., 2022). Another *in vitro* and *in vivo* study indicated that CGA treatment alleviated intestinal inflammation (Xu et al., 2024; Wang et al., 2022). Moreover, CGA can effectively alleviate arthritis symptoms in mice with collagen-induced arthritis (CIA) and inhibit the inflammatory response of MH7A cells by downregulating BAFF expression via inhibition of the NF-κB signaling pathway (Fu et al., 2019).

#### 3.1.2 Inhibition of the MAPK signaling pathway

The mitogen-activated protein kinase (MAPK) signaling pathway plays a key role in the transcriptional regulation of inflammatory mediators. CGA can inhibit the phosphorylation of extracellular signal-regulated kinase (ERK), c-Jun N-terminal kinase (JNK), and p38 MAPK, thereby blocking the transmission of the MAPK signaling pathway and reducing the production of inflammatory mediators (Gao et al., 2019). Inflammatory mediators such as TNF-α, IL-1β, IL-6, prostaglandin E2 (PGE2), and NO play crucial roles in the inflammatory response. CGA exerts anti-inflammatory effects by reducing the expression of inflammatory factors like COX-2 and iNOS, and inhibiting the release mechanisms of pro-inflammatory factors such as TNF-α, IL-1β, and IL-6 (Gu et al., 2023). Furthermore, CGA ester saponins can inhibit the production of PGE2 by suppressing the expression and activity of COX-2, thereby exerting anti-inflammatory effects (Guan et al., 2014).

### 3.2 Regulation of immune cell function

CGA possesses significant regulatory effects on both innate and adaptive immunity, capable of mitigating tissue damage by inhibiting the over-activation, proliferation, and chemotaxis of immune cells.

#### 3.2.1 Inhibition of macrophage and neutrophil activation

CGA exhibits anti-inflammatory activity by inhibiting the activation of various inflammatory cells, including macrophages, neutrophils, and lymphocytes (Han et al., 2023; Chen et al., 2018). Lipopolysaccharide (LPS) can induce inflammatory cells to cause various inflammatory diseases, such as sepsis (Yang TY. et al., 2022), rheumatoid arthritis, and inflammatory bowel disease (Li R. et al., 2022; Cheng et al., 2022). In LPS-induced macrophage inflammation models, CGA can inhibit macrophage proliferation and activation, reducing the expression and secretion of IL-1β, IL-6, TNF-α, COX-2, and iNOS, thereby decreasing their migration to inflammatory sites (Kim et al., 2023).

#### 3.2.2 Regulation of immune recognition and cell migration

CD36 is a scavenger receptor involved in immune recognition and the initiation of inflammatory responses; its upregulation is closely associated with the exacerbation of inflammation. Studies have found that CGA can inhibit the expression of CD36, thereby blocking the migration and infiltration of macrophages and neutrophils into damaged tissues (Marino et al., 2022). This mechanism holds significant clinical potential for the treatment of inflammatory diseases characterized by immune cell infiltration, such as atherosclerosis and arthritis.

### 3.3 Regulation of autophagy

Autophagy is a cellular metabolic process in which cells degrade intracellular damaged organelles and macromolecules via lysosomes to maintain normal physiological activities and homeostasis (Qi et al., 2024). Dysregulation of autophagy function is closely related to inflammatory responses and cell mortality rates; CGA exerts cytoprotective effects by moderately regulating autophagy levels. In a mouse model of mucositis induced by the chemotherapeutic drug 5-fluorouracil (5-FU), CGA was found to upregulate the anti-apoptotic protein Bcl-2 and downregulate the pro-apoptotic molecules Bax and caspase-3, protecting normal tissues such as intestinal epithelial cells and bone marrow hematopoietic stem cells from 5-FU-induced apoptosis. Concurrently, CGA inhibited autophagy activation by downregulating autophagy markers such as LC3-II/I, beclin 1, and p62, ultimately alleviating mucosal injury (Lin et al., 2025). This provides a new therapeutic strategy for alleviating the side effects of chemotherapy.

## 4 Antibacterial effects and mechanisms

CGA exhibits broad-spectrum antibacterial activity, with mechanisms encompassing the disruption of bacterial physical barriers, the regulation of host immune protein function, and the blockade of key inflammatory signaling pathways.

### 4.1 Disruption of cell wall/cell membrane integrity

CGA demonstrates significant inhibitory effects against both Gram-positive (Turumtay et al., 2017) and Gram-negative bacteria (Touzout et al., 2023). At the molecular level, CGA can competitively bind to the active sites of transpeptidases (such as PBP2a), blocking the cross-linking of peptidoglycan chains and leading to defects in the reticular structure of the cell wall (Quintanilla, 2023). Concurrently, CGA targets and disrupts the bacterial cell wall (e.g., the peptidoglycan layer) and induces the overexpression of endogenous lysozymes, accelerating cell wall degradation. This process results in increased membrane permeability and osmotic lysis. *In vivo* pharmacodynamic evaluations using a multidrug-resistant *Escherichia coli* model indicate that CGA effectively inhibits bacterial colonization and biofilm formation, thereby alleviating symptoms of mastitis (Feng et al., 2023).

### 4.2 Inhibition of key protein activity

Beyond direct bactericidal action, CGA exerts anti-inflammatory and antibacterial effects by modulating the phenotype of host immune cells. Using a *Klebsiella* pneumoniae-induced pneumonia mouse model and an *in vitro* polarization model of pulmonary alveolar macrophages (AMs), Li et al. found that CGA promoted the transition of AMs from the pro-inflammatory M1 phenotype to the anti-inflammatory and pro-repair M2 phenotype by increasing SIRT1 expression and enzymatic activity. Mechanistic investigations confirmed that activated SIRT1 acts directly on HMGB1 to inhibit inflammation, thereby effectively alleviating *Klebsiella* pneumoniae-induced pneumonia symptoms and improving the survival rate of infected mice (Li QR. et al., 2022).

### 4.3 Blocking pro-inflammatory signaling pathways

CGA inhibits inflammatory signaling cascades by competitively blocking Toll-like receptors (TLRs) through mimicking the spatial conformation of pathogen-associated molecular patterns (PAMPs). In anti-toxin research, molecular docking and cellular experiments have indicated that lipopolysaccharide (LPS) binds to TLR4 via the CD14/MD-2 complex, inducing TLR4 dimerization, recruiting adaptor proteins MyD88 and TRIF, and activating the TLR4 pathway. The vicinal dihydroxy structure of CGA is spatially similar to the lipid A moiety of LPS, allowing it to competitively bind to the MD-2 protein, blocking the formation of the TLR4-MD-2-LPS complex, and thereby inhibiting the TLR4/NF-κB pathway (Wang et al., 2017). In terms of regulating the anti-tumor microenvironment, cell wall components of *S. aureus* (such as lipoteichoic acid, LTA, and peptidoglycan, PGN) bind to the TLR2-TLR6 heterodimer, activating NF-κB via a MyD88-dependent pathway. This pathway promotes the expression of matrix metalloproteinases (MMPs), enhancing the invasiveness of tumor cells. CGA binds to the leucine-rich repeat (LRR) domain of the TLR2 extracellular domain, inhibiting TLR2-TLR6 heterodimerization and reducing MyD88 recruitment, thereby inhibiting the TLR2/NF-κB pathway and exerting effects in breast cancer (Yang et al., 2022b).

## 5 Antiviral effects and mechanisms

CGA exhibits broad-spectrum antiviral activity by modulating host immune signaling pathways and directly interfering with the viral replication cycle, holding potential for the development of novel antiviral therapeutics (Abomughaid et al., 2022). Regarding hepatitis viruses, CGA demonstrates not only direct antiviral effects but also efficacy in alleviating inflammation-induced tissue damage following viral infection. *In vitro* experiments (HepG 2.2.15 cell model) confirmed that CGA and its related compounds effectively inhibit HBV-DNA replication and the production of hepatitis B surface antigens. Further studies in a duck hepatitis B virus (DHBV) infection model showed that CGA and caffeic acid reduce serum DHBV levels. Concurrently, CGA was shown to inhibit duck viral hepatitis (DEV) by regulating the NF-κB signaling pathway (Yang et al., 2017), indicating the potential of CGA for cross-species inhibition of HBV proliferation. Additionally, the optimization of drug delivery systems has significantly enhanced the efficacy of CGA against coronaviruses. CGA-loaded PVA/PLGA nanoparticles can enhance the antiviral activity of CGA. *In vitro* experiments demonstrate that CGA-loaded PVA/PLGA nanoparticles exhibit significant antiviral activity against coronaviruses and Middle East Respiratory Syndrome Coronavirus (MERS-CoV), with IC50 values significantly lower than those of free CGA (Saleh et al., 2023). These results confirm that PVA/PLGA nanoparticles improve the bioavailability and targeting of CGA, thereby enhancing its antiviral action. Regarding Human Immunodeficiency Virus (HIV), CGA extracted from mulberry bark exhibits anti-HIV activity with low cytotoxicity. Mechanistic studies reveal that CGA primarily blocks viral replication by inhibiting the function of HIV reverse transcriptase. Furthermore, molecular target identification suggests that HCK, EGFR, SRC, and PDPK1 may be potential targets for CGA in inhibiting HIV infection (Li et al., 2021). In the HepG 2.2.15 cell model, CGA and related compounds can inhibit HBV-DNA replication and HBsAg production (Wang et al., 2009), indicating CGA’s potential to suppress HBV proliferation. One of the major derivatives of CGA, 3,4,5-tricaffeoylquinic acid (3,4,5-TCQA), has been shown to specifically inhibit Influenza A Virus (IAV). The TLR3/7 signaling pathway is a critical immune response pathway following IAV infection, participating in inflammatory reactions and antiviral immunity. 3,4,5-TCQA inhibits the production of inflammatory cytokines by suppressing the TLR3/7 signaling pathway, thereby constructing a cellular microenvironment unfavorable for viral replication and effectively inhibiting IAV proliferation (Wang et al., 2024).

## 6 Antifungal effects and mechanisms

Fungal invasion can induce a series of pathological processes in the human body, ranging from local infections and allergic reactions to severe systemic infections and even malignancies (Rinker et al., 2024). Regarding *Candida* albicans, a common clinical pathogen, studies have found that CGA exerts significant antifungal activity through multi-target mechanisms. At the molecular level, CGA downregulates the expression of biofilm-related genes (mrkAD, treC, wbbM) and the quorum sensing gene luxS, thereby interfering with bacterial population behavior. Concurrently, CGA disrupts potassium metabolism, induces apoptosis, and effectively inhibits the transition from yeast to pathogenic pseudohyphae. This efficacy was quantitatively confirmed in vitro experiments: the microbroth dilution method determined the minimum inhibitory concentration (MIC) of CGA against C. albicans to be 80 μg/mL; furthermore, at a sub-inhibitory concentration of 40 μg/mL, CGA significantly disrupted hyphal growth morphology, indicating its strong anti-virulence capacity and effectiveness in damaging hyphal growth (Rajasekharan et al., 2017).

## 7 Regulation of metabolism and mechanisms

Metabolism plays a pivotal role in maintaining human health. However, in both occupational and domestic environments, prolonged sedentary behavior combined with excessive caloric intake has led to a rising prevalence of metabolic disorders. In this context, certain pharmacological agents claiming to regulate metabolism without the need for physical exercise are gaining popularity among young people. It must be emphasized that pharmacological intervention for metabolic disorders should only be initiated when strict clinical indications are met, while lifestyle modifications—including dietary control and regular exercise—remain the cornerstone of metabolic management.

### 7.1 Regulation of glucose metabolism

CGA exhibits a multi-target mechanism in glucose regulation (Yang et al., 2024). It enhances the activity of the insulin signaling pathway by promoting the phosphorylation of Akt, GSK3β, and S6K, which in turn reinforces insulin signaling. CGA possesses antioxidant properties that protect pancreatic β-cells (Ihara et al., 2023). Furthermore, it ameliorates insulin resistance, one of the key pathogenic mechanisms of type 2 diabetes. Mouse glucose tolerance model experiments indicate that CGA regulates glucose and lipid metabolism and improves insulin resistance by activating the AMP-activated protein kinase (AMPK) signaling pathway, promoting glucose uptake and utilization, and inhibiting hepatic glycogenolysis (Ong KhangWei et al., 2012). CGA can also activate the CTRP3-AdipoR2-PPARα signaling pathway, thereby upregulating the expression of hepatic ELOVL6 protein and participating in the regulation of ceramide acyl-chain length, which exerts a dual effect in lowering blood glucose and lipid levels (Guo et al., 2022). Additionally, CGA inhibits protein tyrosine phosphatase 1B (PTP1B) and activates the peroxisome proliferator-activated receptor γ (PPARγ) pathway (Pitschmann et al., 2014). These findings provide a theoretical basis for its application in the treatment of metabolic diseases such as diabetes.

### 7.2 Regulation of lipid metabolism

CGA demonstrates significant activity in regulating cellular energy metabolism and promoting tissue regeneration. In experimental models, researchers have often employed hepatocyte and skeletal muscle cell systems to investigate the biological effects of 100 μg/mL CGA on human embryonic stem cells. Molecular mechanism and metabolic regulation studies indicate that CGA enhances the proliferative capacity of cells (hepatocytes, skeletal muscle cells) by promoting the expression of fatty acid β-oxidation (FAO), thereby facilitating the proliferation and lipid synthesis of human embryonic stem cells (hESCs). Transcriptomic analysis revealed that following CGA treatment, the expression of genes related to glycolysis, the TCA cycle, oxidative phosphorylation (OXPHOS), and one-carbon metabolism was upregulated (Zhao et al., 2023). Notably, targeted metabolomics analysis showed that changes in S-adenosyl-L-methionine (SAM), a product of one-carbon metabolism, were the most significant. Further epigenetic mechanism studies revealed that acetyl-CoA generated from CGA-induced FAO promotes lipid synthesis via H3K27 hyperacetylation and enhances the expression of lipid metabolism-related genes (Zong et al., 2025).

### 7.3 Clinical application of the metabolic regulatory effects of chlorogenic acid

Diabetes often triggers the excessive production of mitochondrial superoxide anions, leading to tissue damage; its core pathological mechanism is the “oxidative stress theory.” Chlorogenic acid protects pancreatic islet β-cells from damage through antioxidant mechanisms and improves insulin resistance (Newsholme et al., 2016). It can inhibit oxidative stress and inflammatory responses in renal tissues, reduce proteinuria, and delay the progression of renal fibrosis. In terms of metabolic syndrome management, numerous short-to medium-term randomized controlled trials (RCTs) have confirmed that chlorogenic acid supplementation significantly lowers blood pressure and improves fasting blood glucose, HOMA-IR index, and blood lipid profiles. Epidemiological evidence supports that chlorogenic acid can slightly reduce body weight and body fat percentage while improving flow-mediated dilation (Zamani et al., 2023). Additionally, multiple cohort studies have found that long-term consumption of coffee rich in chlorogenic acid is significantly negatively associated with the risk of developing type 2 diabetes (Onakpoya et al., 2015).

In the management of type 2 diabetes, clinical medications such as metformin provide potent glycemic control. Direct comparative data indicate that while CGA has significant effects in lowering blood glucose and sensitizing insulin, its efficacy in reducing glycated hemoglobin is generally inferior to that of metformin and sulfonylureas (Asbaghi et al., 2020). However, CGA possesses unique advantages in ameliorating oxidative stress and lipid metabolism disorders; therefore, CGA may serve as an adjunctive therapy to standard hypoglycemic agents, potentially enhancing metabolic regulation and mitigating drug-induced side effects when used in combination.

## 8 Neuroprotective effects and mechanisms

CGA ameliorates neurological deficits and brain edema following intracerebral hemorrhage (ICH). At 24 h and 72 h post-ICH, CGA treatment significantly reduced brain water content and improved outcomes in neurological behavioral tests. *In vitro* experiments have demonstrated that CGA treatment significantly reduces brain edema and improves neurological behavioral outcomes. Mechanistically, CGA inhibits the expression of EMMPRIN and MMP-2/9, thereby attenuating neuroinflammation. This effect is associated with a reduction in microglia/macrophage activation and neutrophil infiltration following ICH. Consequently, CGA treatment mitigates neuronal cell death and brain injury (Liu et al., 2022). Studies indicate that CGA inhibits hypoxia-reoxygenation (OGD/R)-induced apoptosis in human brain microvascular endothelial cells (HBMECs) by activating the PI3K-Akt signaling pathway. Furthermore, CGA reduces cerebral infarction volume and neuronal apoptosis induced by middle cerebral artery occlusion (MCAO) in mice and promotes angiogenesis (Fan et al., 2022).

CGA increases the levels of cyclic guanosine monophosphate (cGMP) in diabetic hearts by promoting the production of nitric oxide (NO). CGA intervention significantly increases the phosphorylation level of endothelial nitric oxide synthase (eNOS), enhancing its activity and thereby increasing NO generation. The elevated NO activates soluble guanylate cyclase (sGC), ultimately leading to elevated cGMP levels (Qin et al., 2021).

### 8.1 Clinical application of the neuroregulatory effects of chlorogenic acid

The pathogenesis of Alzheimer’s disease (AD) and Parkinson’s disease (PD) is closely related to elevated levels of oxidative stress in the brain. Due to its high oxygen consumption and high lipid content, brain tissue is highly susceptible to free radical attack (Butterfield and Boyd-Kimball, 2018). Chlorogenic acid exhibits significant neuroprotective effects; it is capable of crossing the blood-brain barrier, scavenging free radicals within the brain, and inhibiting the aggregation and oxidation of β-amyloid (Aβ) (Lakey-Beitia et al., 2015). Currently, relevant research is primarily in the preclinical and clinical trial stages, and chlorogenic acid is regarded as a potential dietary supplement for the prevention and delay of AD and PD progression. Furthermore, epidemiological cohort studies have observed that long-term intake of beverages rich in chlorogenic acid is positively correlated with a reduced risk of PD and AD (Larsson et al., 2022), providing population-level support for the hypothesis that chlorogenic acid protects neurons through antioxidant and anti-inflammatory mechanisms.

Current treatment regimens for AD and PD primarily rely on cholinesterase inhibitors and levodopa, respectively, focusing mainly on symptom management without altering the underlying pathological mechanisms of the diseases. To date, no large-scale clinical trials have been conducted. Preclinical data indicate that direct comparisons between CGA and these standard neuroprotective or symptomatic relief drugs show that CGA exhibits efficacy comparable to certain neuroprotective agents in animal models. However, unlike standard drugs that provide immediate symptomatic relief, the therapeutic value of CGA lies primarily in preventive interventions and delaying disease progression through long-term antioxidant and anti-inflammatory mechanisms.

## 9 Antitumor effects and mechanisms

Extensive evidence-based medical research based on *in vivo* and *in vitro* cancer models indicates that CGA is capable of reducing the incidence of chemical carcinogenesis and demonstrates broad-spectrum antitumor bioactivity (Huang et al., 2019). Its anticancer mechanisms involve multiple aspects, including inducing tumor cell differentiation, promoting tumor cell apoptosis, regulating macrophage polarization, and inhibiting tumor cell glycolysis. Regarding drug combination therapy and microenvironment modulation, Li et al. found that the traditional Chinese medicine compound RLT-03 (containing Astragaloside IV and CGA) exerts antitumor effects by inhibiting the expression of VEGF, EGF, IL-10, TGF-β, and CD34 in the tumor microenvironment, thereby blocking receptor tyrosine kinase signaling and ameliorating the immunosuppressive microenvironment. This mechanism effectively inhibited the proliferation and migration of breast cancer cells and induced apoptosis (Li et al., 2020).

In vivo and *in vitro* studies targeting human glioma U373 cells, the research team adopted a comprehensive strategy of “network pharmacology prediction - *in vitro* cell experiments - *in vivo* animal model validation.” CGA downregulated the expression and phosphorylation levels of SRC and MAPKs proteins in U373 cells, thereby inhibiting their proliferation, migration, and invasion, and inducing apoptosis and cell cycle arrest. CGA inhibited U373 cell growth in nude mice and induced apoptosis. Network pharmacology analysis predicted that the SRC/MAPKs signaling pathway is associated with the antitumor effects of CGA (Zhou et al., 2021). These studies indicate that CGA demonstrates tremendous translational potential in the oncology field through the precise regulation of various molecular mechanisms, providing an important experimental basis for new drug development.

## 10 Regulatory effects of chlorogenic acid on the gut-brain axis

Increasing evidence underscores the gut-brain axis as a pivotal signaling pathway connecting the enteric nervous system (ENS) with the central nervous system (CNS), playing a fundamental role in the maintenance of physiological homeostasis. The gut-brain axis represents a complex interactive network involving the gut, the nervous system, and the immune system. It conveys information through neural, endocrine hormonal, and immune signaling, profoundly influencing brain function and emotional states. As a prominent dietary polyphenol, CGA has demonstrated substantial potential in modulating this bidirectional communication network.

### 10.1 Regulation of gut microbiota by chlorogenic acid

The gut microbiota maintains a dynamic symbiotic relationship with the host and dietary intake. As a complex ecosystem within the gastrointestinal tract, it functions as a crucial metabolic pathway, transforming indigestible dietary components into bioactive metabolites. This tripartite interaction is paramount in determining host metabolic homeostasis. Consequently, the gut microbiota serves as a pivotal bridge between dietary intake and host physiology (Shi et al., 2021). Within the context of the gut-brain axis, CGA, owing to its polyphenol structure, exhibits potent prebiotic-like properties, significantly modulating the composition and metabolic activity of the gut microbiota. Upon ingestion, CGA and its metabolites selectively promote the proliferation of beneficial bacteria, such as *Lactobacillus* and Bifidobacterium, while concurrently inhibiting the growth of pathogenic bacteria. This regulation of microbial ecology fosters a favorable intestinal environment. Furthermore, CGA undergoes biotransformation by the gut microbiota into bioactive metabolites, including acetate, propionate, and butyrate–the short-chain fatty acids (SCFAs). These SCFAs not only serve as energy sources for colonocytes but also act as key signaling molecules, mediating systemic anti-inflammatory and neuroprotective effects, thereby effectively bridging intestinal health and brain function within the gut-brain axis (Guo et al., 2025; Zhang et al., 2022).

### 10.2 Gut-brain axis signaling

The interaction of CGA with the gut-brain axis is mediated through a complex signaling network, primarily involving the vagus nerve, immune modulation, and circulating metabolites (Zhu H. et al., 2024). CGA and its microbiota-derived metabolites can stimulate afferent vagal nerve fibers, transmitting signals directly to the brainstem, subsequently reaching higher brain centers, thereby influencing autonomic and neuroendocrine functions (Bhattacharjee et al., 2022). Furthermore, short-chain fatty acids produced through CGA biotransformation, particularly butyrate, act as histone deacetylase (HDAC) inhibitors, modulating gene expression in neurons and glial cells, thus introducing an epigenetic layer to gut-brain communication. In terms of immune regulation, CGA modulates intestinal immune responses by inhibiting key signaling pathways such as NF-κB and MAPK, reducing the production of pro-inflammatory cytokines (e.g., TNF-α, IL-6), and alleviating systemic low-grade inflammation–a known contributor to neuroinflammation. Critically, CGA enhances the integrity of the intestinal barrier and the blood-brain barrier (BBB). By upregulating the expression of tight junction proteins (e.g., ZO-1, occludin), CGA prevents the translocation of bacterial toxins (e.g., lipopolysaccharide) into circulation, thereby protecting the brain from peripheral inflammation and maintaining neural homeostasis (Xue et al., 2023; Zhang B. et al., 2024).

### 10.3 Neuroregulation

CGA-mediated modulation of the gut-brain axis translates into profound neuromodulatory effects, influencing mood, cognitive function, and stress resilience. By modulating the gut microbiota and the subsequent generation of neuroactive metabolites, CGA impacts the synthesis and conversion of key neurotransmitters in the brain, including serotonin, dopamine, and brain-derived neurotrophic factor (BDNF) (Hung et al., 2021). This modulatory mechanism forms the basis for CGA’s potential therapeutic efficacy in neuropsychiatric and neurodegenerative disorders. Notably, recent studies, including the work by Zhang et al., have demonstrated significant neuroprotective effects of CGA, potentially involving the mitigation of neuroinflammation and oxidative stress, thereby highlighting its potential in the therapeutic management of complex neurological diseases (Hou et al., 2023). Preclinical evidence indicates that CGA administration improves depressive-like and anxiety-like behaviors in animal models, possibly by normalizing hypothalamic-pituitary-adrenal (HPA) axis activity and reducing oxidative stress in neural tissues (Xie et al., 2024). Furthermore, emerging evidence suggests that CGA enhances cognitive performance and memory retention, likely mediated through its anti-inflammatory properties in the hippocampus and its ability to ameliorate deficits in synaptic plasticity (Lee et al., 2020).

## 11 Epigenetic mechanisms

In recent years, epigenetics has emerged as a new Frontier in the study of drug action mechanisms. CGA has also been demonstrated to precisely regulate gene expression networks at the post-transcriptional level by intervening in DNA methylation, histone modifications, and the expression of non-coding RNAs (such as microRNAs). This modulation enables CGA to regulate key biological signaling pathways, thereby explaining its broad pharmacological activities, ranging from anti-inflammatory to neuroprotection (Li, 2021). Notably, recent research has further emphasized the central role of DNA methylation within this regulatory framework, establishing CGA’s status as a natural agent capable of reshaping the epigenetic landscape and subsequently restoring cellular homeostasis.

### 11.1 Regulation of DNA methylation

Emerging evidence suggests that the therapeutic potential of CGA extends beyond traditional signaling pathways to encompass epigenetic regulation, particularly DNA methylation. As a natural epigenetic modulator, CGA can influence the activity of DNA methyltransferases (DNMTs) and the metabolism of methyl donors. In the context of cancer prevention and treatment, CGA has been shown to inhibit tumor cell proliferation and induce apoptosis by reversing aberrant DNA hypermethylation states, thereby reactivating the expression of silenced tumor suppressor genes (Hernandes et al., 2020). Furthermore, recent studies indicate that CGA can modulate DNA methylation patterns in the brain, hinting at potential neuroprotective mechanisms through the epigenetic regulation of genes involved in neuroplasticity and inflammation. This epigenetic reprogramming capability provides a novel theoretical basis for the clinical application of CGA in epigenetics-related diseases, such as cancer and neurodegenerative disorders.

### 11.2 Regulation of histone modification

Histone modification is another important pathway by which DNA regulates gene expression. Current research not only focuses on the impact of CGA on histone modifications under pathological states but also involves the epigenetic regulatory basis during its biosynthesis. For example, in research involving the plant source *Lonicera japonica*, treatment with the DNA methyltransferase inhibitor 5-azaC significantly altered the expression profile of histone methyltransferases (e.g., decreasing HMT1/4, increasing HMT2/3/5) and led to extensive remodeling of histone methylation marks (H3K4me3, H3K9me1/2/3). Changes in this “histone code” were closely correlated with the accumulation of active ingredients: levels of H3K4me2/3 and H3K9me3 were positively correlated with the content of flavonoids and iridoids (Liu et al., 2020). This suggests that specific histone modification sites in plant cells are key molecular switches regulating the synthesis of secondary metabolites (including CGA and its synergistic components). Although this belongs to plant physiological mechanisms, it reveals the epigenetic origins of the production of active ingredients like CGA. In mammalian models, similar mechanisms suggest that CGA may exert anti-inflammatory and antitumor effects by regulating specific histone acetylation or methylation levels, thereby controlling the chromatin accessibility of inflammatory factors or apoptosis-related genes (Liu et al., 2020).

### 11.3 Regulation of MicroRNA (miRNA) expression

CGA can also block disease signaling pathways by upregulating or downregulating the expression of specific miRNAs. Studies indicate that CGA can target and regulate key miRNAs related to the cell cycle, angiopoietins, and immune response (such as miR-21, miR-34, etc.), forming a “CGA-miRNA-mRNA” regulatory axis. This mechanism not only enhances the precision of regulation for downstream target genes (such as PTEN, p53, and NF-κB) but also provides potential strategies for developing CGA-based miRNA-targeted therapies (Zhang et al., 2023; Roshan‐Milani et al., 2022).

## 12 Other biological activities and mechanisms

CGA possesses various other potential health benefits. Research indicates that CGA exhibits anti-complement effects (Luo et al., 2020), regulates immune system function (Wang et al., 2025), protects nerve cells from damage, and protects the liver from toxic substances (Yao et al., 2025). These research results suggest that CGA has broad application prospects in maintaining human health and is worthy of further research and development. Specific mechanisms are shown in (Table 1).

**TABLE 1:** The biological activity of CGA and its molecular mechanism of action.

Biological activity	Molecular mechanism	Experiment type	References
Neuroprotective effect	It can reduce oxidative damage to nerve cells and has potential protective effects on neurodegenerative diseases such as Alzheimer’s disease and Parkinson’s disease; Inhibiting excessive activation of microglia, thereby reducing neuroinflammatory responses.	Animal Cells experiment	Shi et al. (2023), He et al. (2023), Shen et al. (2012)
Liver protection effect	Inhibiting the activation of hepatic stellate cells, reducing collagen deposition, and delaying the process of liver fibrosis; By regulating lipid metabolism, reducing liver fat accumulation, improving fatty liver.	Animal experiment	Moslehi et al. (2023)
Anti-osteoporosis effect	By promoting osteoblast differentiation and inhibiting osteoclast activity to improve bone density.	Clinical trial	Jiang et al. (2024)
Regulate the intestinal flora	The metabolism of intestinal microorganisms produces active components such as caffeic acid and ferulic acid, regulating the balance of intestinal flora, improving intestinal barrier function, and having potential benefits for inflammatory bowel disease (IBD).	Animal experiment	Zeng et al. (2024)
Radiation-proof effect	Reducing DNA damage and cell apoptosis caused by ionizing radiation, which has a certain effect on radiation protection such as radiotherapy side effects.	Cells experiment	Yin et al. (2022)
Promote wound healing	Promoting fibroblast proliferation and collagen synthesis, accelerating skin wound repair. Promoting the migration and closure of wounds. Promoting the formation of capillaries in human umbilical vein endothelial cells.	Cells experiment	Moghadam et al. (2017)
Regulate the metabolism of trace elements	Affecting the concentrations of trace elementssuch as iron and zinc in plasma, which may have regulatory effects on anemia and diseases related to trace element deficiency.	Experiment on microbiology	(Gandhi et al., 2023)

## 13 Research on the drug metabolism system of chlorogenic acid

Although CGA possesses various biological activities such as antioxidant, anti-inflammatory, antibacterial, and anti-gout effects, in practical applications, it also has disadvantages such as instability, rapid metabolism, and short half-life due to the dual limitations of poor solubility and rapid *in vivo* metabolism (Clifford et al., 2020). Pharmacokinetic (PK) studies have quantitatively revealed the root causes of its low bioavailability: after oral administration, the peak plasma concentration time (Tmax) of CGA is relatively short (usually 0.5–1 h), and the absolute bioavailability is low (usually <10% in rat models, with some studies even showing <0.2%–1%). More critically, the elimination half-life (t1/2) of CGA in plasma is very brief (approximately 0.5–2 h), leading to insufficient retention time of the drug in the body and difficulty in maintaining an effective therapeutic concentration (Zhu et al., 2013). Therefore, a delivery system or combination with other technologies is needed to enable CGA to function better. CGA is mainly absorbed in the small intestine, and its absorption efficiency is influenced by the formulation, pH value, and gut microbiota. It is mainly distributed in organs such as the liver, kidneys, heart, and brain, and is metabolized by the liver into products such as caffeic acid, quinic acid, and protocatechuic acid (Shen et al., 2022). Due to the hydrophilicity of CGA, only a limited amount of CGA can be absorbed by gastrointestinal epithelial cells and enter the systemic circulation after the administration of a certain dose. About one-third of CGA can be metabolized (Chen et al., 2017). In experiments, high doses of CGA or combination with other technologies are required.

## 14 Different types of drug delivery systems

### 14.1 Polymer nanodrug delivery systems

With their good biocompatibility, degradability, and ease of functionalization, polymer nanoparticles have become important carriers for CGA delivery systems. Through physical encapsulation or chemical conjugation, this system can effectively solve clinical application bottlenecks such as the susceptibility of CGA to photothermal degradation, short half-life, and low bioavailability (Lin et al., 2024). In the antitumor field, nanocarriers can also significantly improve the tumor targeting and enrichment capability of drugs. For example, Buskaran et al. developed a graphene oxide (GO) nanocomposite loaded with catecholamine and CGA for the treatment of human hepatocellular carcinoma. Studies showed that the nanocomposite had lower cytotoxicity to normal cells and significant cytotoxicity to liver cancer cells; the synergistic effect of the dual drugs enhanced anticancer activity, could be effectively absorbed by liver cancer cells, induced late apoptosis, and blocked the G2/M cell cycle. This further led to the depolarization of mitochondrial membrane potential and the production of intracellular reactive oxygen species, triggering apoptosis (Buskaran et al., 2021). Another study indicated that glioblastoma is the most aggressive brain tumor with a poor prognosis. Tumor-associated macrophages (TAMs) usually exhibit an immunosuppressive phenotype, typically the M2 phenotype. Ye et al. constructed mannosylated liposomes (based on DSPE-PEG2000-mannose) to utilize the active targeting of mannose receptors to tumor-associated macrophages (TAMs) for the precise delivery of CGA. This promoted their polarization from M2 to M1, thereby exerting antitumor effects. This further inhibited the growth of G422 glioblastoma (Ye et al., 2020), providing new ideas for the immunotherapy of refractory brain tumors.

### 14.2 Liposome drug delivery systems

Due to their lipid-like molecular layer structure being similar to the cell membrane, liposomes possess excellent biocompatibility and can effectively improve the solubility of poorly soluble drugs and ameliorate *in vivo* distribution. Rui et al. established a sustained-release drug system by encapsulating CGA in sustained-release materials such as polymers or liposomes, extending its retention time *in vivo* and overcoming the limitation of rapid elimination of CGA, thus providing a feasible solution for maintaining long-term effective blood concentrations (Rui et al., 2025). To further activate the antitumor immune response, (Ye et al., 2021) explored the antitumor immunotherapeutic efficacy of CGA using a self-microemulsifying drug delivery system (SMEDDS). Research results showed that CGA-SMEDDS was effective in efficiently activating antitumor immune responses and inhibiting tumor growth by promoting drug accumulation in mesenteric lymph nodes (MLNs). CGA-SMEDDS not only promoted dendritic cell maturation and T cell activation but also inhibited immunosuppressive components, thereby effectively inhibiting tumor growth and inducing long-term immune memory effects, which has important clinical significance for preventing postoperative tumor recurrence and metastasis (Ye et al., 2021).

### 14.3 Microsphere drug delivery systems

The microsphere drug delivery system mainly uses biodegradable polymer materials such as PLGA and chitosan as skeletons to disperse or encapsulate the drug, forming micron-sized spherical carriers. The core clinical value of this system lies in achieving long-acting sustained release and local positioning, making it particularly suitable for chronic diseases requiring long-term administration or local treatment (Fang et al., 2023). In the treatment of joint diseases such as osteoarthritis, studies have shown that after intra-articular injection of CGA microspheres, the burst release effect of the drug can be significantly reduced, maintaining an effective drug concentration in the joint cavity for several weeks, thereby persistently inhibiting the expression of inflammatory factors and protecting the cartilage matrix, avoiding the gastrointestinal side effects brought by frequent high-dose use of traditional oral administration. In the field of oral administration, microspheres can protect CGA from degradation by the harsh gastrointestinal environment and achieve release at specific sites in the intestine by regulating the particle size, further improving its oral bioavailability (Zhang Y. et al., 2024). It avoids gastric mucosal injury caused by high oral doses (>500 mg/kg) (reduced histological scores). This provides a highly potential formulation strategy for the long-term management of chronic inflammatory diseases with CGA.

#### 14.3.1 Comparison of the advantages of various drug delivery systems

Among the various strategies aimed at improving the delivery efficiency of CGA, nanoparticles, liposomes, and microspheres each possess distinct advantages, though their application scenarios differ significantly. Comparative studies indicate that polymer nanoparticles typically exhibit the most superior performance in enhancing oral bioavailability. This is attributed to their smaller particle size, which facilitates intestinal lymphatic uptake, thereby circumventing hepatic first-pass metabolism; some studies suggest that, compared to native CGA, nanocarriers can increase bioavailability by several folds (Fu et al., 2024). In contrast, due to the similarity of their bilayer structure to biological membranes, liposomes possess unique advantages in enhancing cell membrane penetration and improving *in vivo* tissue distribution, demonstrating significant potential for crossing the blood-brain barrier, particularly for brain-targeted delivery (Chetehouna et al., 2024). On the other hand, microsphere technology occupies a dominant position in subcutaneous or intramuscular injection therapies requiring sustained release, owing to its long-acting release characteristics, which can prolong the duration of drug action (Chahardoli et al., 2023). In summary, polymer nanoparticles are often selected with the goal of maximizing oral bioavailability; whereas, if the objective is “specific tissue (e.g., brain) targeting” or “long-acting sustained release,” functionalized liposomes or microsphere systems offer greater advantages.

#### 14.3.2 Applications of chlorogenic acid delivery systems

By adopting advanced strategies such as nanoparticles, liposomes, micelles, microspheres, cocrystals, and prodrugs, CGA delivery systems effectively overcome inherent shortcomings such as poor solubility, low stability, limited bioavailability, and potential systemic side effects. This enables CGA to reach target sites—such as tumor cells, inflamed areas, or damaged skin tissues—with unprecedented efficiency and precision. Consequently, CGA demonstrates immense potential in key therapeutic areas including oncology (Zhu S. et al., 2024), diabetes (Cheng et al., 2025) and its complication management, neuroprotection, anti-inflammation (Vyawahare et al., 2024), and dermatology (Zhang R. et al., 2024). These delivery systems not only achieve more efficient, precise, and safer targeted therapies.

## 15 Discussion

CGA) is a polyphenolic compound widely found in plants and has attracted significant attention due to its remarkable biological activities, including antioxidant, anti-inflammatory, antibacterial, antitumor, and metabolic regulation effects. This review systematically elucidates the therapeutic mechanisms of CGA, which involve regulating key signaling pathways such as Nrf2, NF-κB, and MAPK, scavenging free radicals, chelating metal ions, inhibiting the release of inflammatory mediators, and modulating immune cell function. Although CGA shows immense potential *in vitro* and *in vivo*, its clinical application is severely restricted by drawbacks such as chemical instability, low oral bioavailability, and rapid metabolism.

In recent years, the development of nano- and micro-delivery systems has significantly improved the stability, targeting, and sustained-release properties of CGA. Particularly in fields such as oncology, neuroprotection, metabolic diseases, and anti-infective therapy, novel delivery strategies have effectively ameliorated the pharmacokinetic properties of CGA, demonstrating broad application prospects. However, while affirming these technological advances, a critical evaluation of the existing literature reveals a significant gap: the evidence derived from *in vitro* (cellular) experiments and animal models is robust, whereas the clinical data currently available is relatively limited.

Among the various biological activities summarized, the blood glucose-regulating effect of chlorogenic acid—particularly in the management of type 2 diabetes (T2DM) and metabolic syndrome—appears to possess the strongest clinical evidence support. Although preclinical models have extensively elucidated its molecular mechanisms, such as inhibiting α-glucosidase, regulating glucose-6-phosphatase translocation, and activating the AMP-dependent protein kinase (AMPK) pathway, clinical trials have moved beyond mechanistic speculation to confirm specific health outcomes. Human randomized controlled trials (RCTs) report that chlorogenic acid supplementation can significantly reduce postprandial blood glucose and glycated hemoglobin (HbA1c) levels and improve insulin sensitivity. This indicates that a translational bridge indeed exists between the molecular pathways observed in rodents and the metabolic phenotypes observed in patients.

Conversely, although the literature regarding the neuroprotective and anticancer properties of chlorogenic acid is extensive in cellular and animal models—with mechanisms involving NF-κB inhibition, Nrf2 activation, and mitochondrial-dependent apoptotic pathways well-established—the clinical evidence in these fields remains preliminary. Most human studies regarding neuroprotection are currently limited to observational epidemiological studies linking high CGA intake, for example, through coffee consumption, to a reduced risk of neurodegenerative diseases, rather than interventional experiments proving causality. While nano-delivery technology shows great potential in enhancing the bioavailability and targeting efficiency of CGA in murine tumor models, its application in human oncology is still in its infancy, with pharmacokinetic profiles and safety yet to be fully established.

Furthermore, when deeply evaluating the extensive pharmacological activities of chlorogenic acid, one must be vigilant regarding the potential for false positives in various biochemical assays. Due to its polyphenolic structure, CGA is a potent reducing agent and free radical scavenger. This chemical nature makes it highly prone to causing non-specific interference in various assay forms *in vitro*. For instance, in enzyme activity assays, CGA can directly reduce chromogenic substrates such as MTT, XTT, and DPPH, or non-covalently bind to enzyme proteins, thereby altering enzyme conformation. These effects do not necessarily represent a specific pharmacological inhibitory effect on a particular biological target. Similarly, in fluorescence or absorbance spectroscopic analyses, the intrinsic fluorescence or quenching effects of CGA may be misinterpreted as signal changes. If appropriate control experiments for these interfering factors—such as excluding spectral interference or using enzyme-inactive controls—are not implemented, relying solely on routine biochemical assay kits may lead to a misinterpretation of the molecular mechanisms of chlorogenic acid. Therefore, future research verifying the interaction of chlorogenic acid with specific targets should rely more on cellular-level functional validation or more rigorous methods based on biomembrane interaction technologies to exclude false positives caused by non-specific redox interference.

In conclusion, while *in vitro* and *in vivo* data provide a compelling basis for CGA as a multifunctional therapeutic agent, and the introduction of nanotechnology has further optimized its delivery strategies, its clinical validation is currently most robust only in the field of metabolic health. To promote the clinical translation of CGA in other areas, future research should prioritize high-quality, large-scale clinical trials. Simultaneously, regarding mechanism exploration, greater reliance should be placed on cellular-level functional validation or more rigorous methods based on biomembrane interaction technologies to eliminate false positives caused by non-specific redox interference, thereby providing a more solid and reliable scientific basis for the clinical application of chlorogenic acid.
